# Deploying the Mental Eye

**DOI:** 10.1177/2041669515607710

**Published:** 2015-10-13

**Authors:** Jan Koenderink, Andrea van Doorn, Johan Wagemans

**Affiliations:** Laboratory of Experimental Psychology, Department of Brain & Cognition, University of Leuven (KU Leuven), Belgium; Faculteit Sociale Wetenschappen, Psychologische Functieleer, Universiteit Utrecht, the Netherlands; Faculteit Sociale Wetenschappen, Psychologische Functieleer, Universiteit Utrecht, the Netherlands; Laboratory of Experimental Psychology, Department of Brain & Cognition, University of Leuven (KU Leuven), Belgium

**Keywords:** mental eye, pictorial relief, viewing modes, shape, depth

## Abstract

Three observers performed a task designed to quantify their “pictorial relief” in visual awareness for a photograph of a piece of sculpture. In separate sessions, they were instructed to assume one of two “mental viewpoints.” The main objective was to investigate whether human observers have such command. All three observers could redirect their “mental view direction” by up to 20°. These observers experience “paradoxical monocular” stereopsis, whereas a sizable fraction of the population does not. Moreover, they had some experience in assuming various “viewing modes.” Whereas one cannot generalize to the population at large, these findings at least prove that it is possible to direct the mental viewpoint actively. This is of importance to the visual arts. For instance, academic drawings require one to be simultaneously aware of a “viewing” (for the drawing) and an “illumination direction” (for the shading). Being able to mentally deploy various vantage points is a crucial step from the “visual field” to the “visual space.”

## Introduction

In matters of vision research, pictures have the decisive advantage that they are undisputedly flat (two-dimensional [2D]) objects. Thus, “pictorial spaces” as are sometimes reported by observers purportedly “looking into” pictures have to be phantasms. After all, “space” apparently refers to some additional degree of freedom (three-dimensional [3D]), whereas pictures are flat (2D). Hence the fact that such reports keep coming in steadily—indeed, are routinely used in the visual arts community—has led to technical jargon such as “inverse optics” (Poggio, Torre, & Koch, 1985) and “paradoxical monocular stereopsis” (Claparède, 1904; Schlosberg, 1941).

An inverse optics algorithm is supposed to yield a 3D scene description for a picture as input. This is technically impossible.^1^ In contradistinction, paradoxical monocular stereopsis is at least a phenomenological fact (Koenderink, van Doorn, & Wagemans, 2011; Schlosberg, 1941), although science (e.g., computer vision) has no explanation for it. An account in terms of experimental phenomenology is desirable.

This article focusses on some aspects of stereopsis (we will drop the “paradoxical” and “monocular” for brevity), which is thus of a phenomenological nature. We start by reminding the reader of some basic facts and concepts (for more background on terminology, see Koenderink, van Doorn, & Wagemans, 2015). Then we discuss novel data and conclude by proposing some concepts that might prove useful in dealing with “pictorial space” and “depth” (Koenderink et al., 2011).

**Figure 1.**
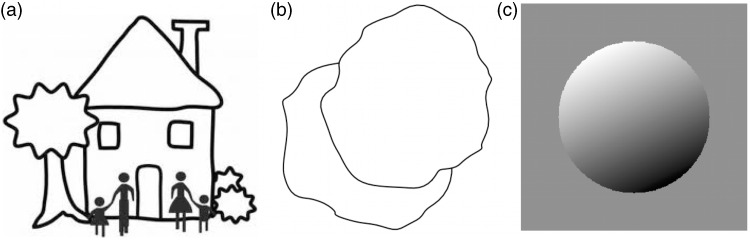
(a) The tree and the people are in front of the house. (b)The top-right blob is in front of the bottom-left one. There is no need for pictorial space here. (c) The “3D sphere” is actually a 2D circular disk filled with a uniform gradient of gray tone. Some people are aware of the former, some of the latter (Metzger, 1975; Ramachandran, 1988; van Doorn, Koenderink, Todd, & Wagemans, 2012; Wagemans, van Doorn, & Koenderink, 2010). Convex–concave flips are only experienced by the former group.

Many, though by no means all, human observers experience “pictorial spaces” when looking into pictures, say holiday snapshots. Probably no one experiences a pictorial space when viewing a picture upside down. This is a common technique used by painters to experience a work as a flat array of pigments, without intrusion of perceptual objects due to stereopsis. Notice that 2D pictures may permit various 3D inferences even when there is no such a thing as “pictorial space.” In Figure 1(a), one immediately “sees” the tree in front of the house. It works just as well for random blobs (Figure 1(b)). No need for 3D phantasms, and a simple 2D algorithm achieves this. The case of Figure 1(c) is perhaps different. It has been used often in vision research (Metzger, 1975; Ramachandran, 1988). Textbooks tell you that you are supposed to “see a sphere,” that is a pictorial, volumetric object. Indeed, many observers do, but some do not (van Doorn et al., 2012; Wagemans et al., 2010). The latter see “a circular disc filled with a linear gradient of gray tone,” as an editor of one of our papers once reminded us. He had never experienced a 3D impression with such stimuli. We would not know how to call him wrong. Various 2D structures allow observers to participate in many “shape from shading” experiments without ever being aware of a 3D shape. We find, however, that persons who experience the 3D impressions have a decisive speed advantage in some paradigms apparently because they simply act on their immediate visual awareness, where the others are faced with a cognitive judgment. Another difference is that the 3D-sensitive observers occasionally experience sudden depth reversals and experience these as remarkable happenings, whereas the 2D observers are never aware of such spooky events. This suggests that one might differentiate between such “viewing modes” (we use “mode” simply to indicate a variety of visual experiences) on objective criteria. Perhaps unfortunately, the 3D observers cannot explain the qualities of awareness experienced by them to the 2D observers because, after all, the picture never changes.

Thus, phenomenologically, human visual awareness comes in diverse “modes.”^2^ At least some observers appear to be able to voluntarily switch modes, others to be simultaneously aware of distinct modes. There appears to be no generally accepted taxonomy of this.

Those who are aware of pictorial space—we estimate possibly more than half of the generic population^3^—describe it as a remarkable mode of awareness. For many visual artists, it is simply part of “being able to see.” Vision scientists who discovered it describe it as remarkable because they know it to be impossible. One very clear report (in only five pages of plain text) is by Harold Schlosberg. Schlosberg fails to understand how such a major visual faculty could remain unknown to his colleagues. He wrote this in 1941. Schlosberg reminds the reader that this was common knowledge in the period around 1910 when people noticed that you really do not need a stereoscope—of course, they meant a binocular one!—to obtain the “plastic effect.” Indeed, it was so throughout the second half of the 19th century.^4^ It has evidently been forgotten now, at least in the sciences. According to Schlosberg, “it should be stressed that the plastic depth that can be obtained monocularly is very striking, and must be seen to be appreciated” (p. 601).

This is still as true as it ever was, indeed a self-evidence in the visual arts, though generally ignored by science.

Over the past two decades, we spent much effort to study these remarkable phenomena, both empirically (Koenderink & van Doorn, 1995; Koenderink & van Doorn, 2003; Koenderink, van Doorn, & Kappers, 1992, 1994, 1996; Koenderink, van Doorn, & Wagemans, 2012; Koenderink, Wijntjes, & van Doorn, 2013; Koenderink, van Doorn, Christou, & Lappin, 1996a, 1996b; Koenderink, van Doorn, Kappers, & Todd, 2001, 2004; Koenderink et al., 2011; van Doorn, Koenderink, & Wagemans, 2011) and formally (Koenderink & van Doorn, 1998, 2012), being convinced that they are of key importance to the understanding of the psychogenesis of visual awareness. We cannot fully summarize the results here, but we mention a few issues that are of generic conceptual interest. Of course, we can offer no “final answers”; the topic remains still pretty much an unexplored territory.

One of the least understood issues involves the relation between scene (in the case there is one), the eye (we mean the optical center of the anatomical structure), the picture (as a planar surface covered with pigments in some particular order), and the awareness of pictorial relief. The first point to grasp here is that the scene does not truly belong in this list. For all one knows, the picture might have been found in a dung hill caused by some peculiar dark fungus growth. This is important because it shows up the notion of “veridicality”—which unfortunately dominates the literature—as irrelevant to the present discussion (Koenderink & van Doorn, 1995; Koenderink et al., 1992, 1994, 2011). The next important point is to understand that pictorial space and the space the observer moves in (Koenderink et al., 2004; von Hildebrand, 1893) are categorically disjunct. Thus, there are no geometrical relations between the eye and pictorial space, as there are none between the eye and the picture (Koenderink et al., 2011). As you view an en face portrait obliquely, the pictorial person looks straight at you (Koenderink et al., 2004). This is often spontaneously noticed (and experienced as “eerie”) by naive observers. Then there is no such a thing as the distance of some pictorial object to your eye. What we call “depth” does not have an origin at the eye, nor does the picture surface have a depth. All you can judge are “ratios of depth differences” of pictorial objects, at least in the simplest instances (Koenderink et al., 2011).

**Figure 2.**
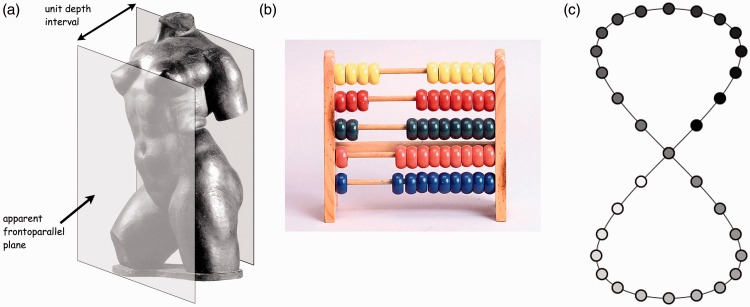
(a) Hildebrand's geometrical idea of pictorial space. He conceives of pictorial objects as sandwiched between two glass panes, touching the object at both extremities. The object is experienced as “living in a depth slice,” thus “unifying awareness of the volume.” This “relief,” or (2 + 1)D, understanding of pictorial space was criticized by some contemporaries as “flat sculpture being like wooden iron.” Hildebrand saw it as “Michelangelo's method (as famously described by Vasari, 1550).” (b)An abacus. It is suggested as a heuristic device to intuit the structure of pictorial space. The wires represent the depth domains, there is one for each location in the picture. Usually there will be just a single bead on a wire, although there can be more. Examples include cases of phenomenal transparency and T-junctions (Metzger, 1975). (c) Such points at different depths at the same location in the picture plane are “parallel points” in the formal geometry. This is shown at right. The point at the central intersection is white, black, and gray, three “parallel points.”

The first theory of pictorial space was formulated by the German sculptor Adolf von Hildebrand in 1893. Throughout the years, we have extended his ideas and formulated them mathematically (Koenderink & van Doorn, 2012). A simplified and somewhat informal account runs as follows. Pictorial space is best understood as (2 + 1)D instead of 3D, say the visual field (for simplicity parameterized by the picture surface) and the depth domain. The depth domain has no correlate in the physical world (Koenderink et al., 2011). It is an ill-defined one-dimensional space, perhaps structurally similar to the affine line.^5^ Pictorial space is constructed by attaching a depth space to each point of the picture plane, all these depth spaces being mutually independent.^6^ As a heuristic, one thinks of these depth domains as threads on which psychogenesis may shift “depth beads” (Figure 2). In an intuitive picture, let all these threads be mutually parallel and at right angles to the picture plane: These are irrelevant embellishments but help “fill out” the mental picture, which looks much like a (2 + 1)D abacus; a standard abacus implements a (1 + 1)D space.

Phenomenologically, these threads are somehow coordinated by the mind. Here Hildebrand has a useful heuristic. He conceives parts of pictorial space as being enclosed by a pair of glass panes. His object to be sculptured would touch the panes at its near and far extremities.

The panes cut through all the wires and thus define points on them. One pane defines points of “zero distance,” the other points of “unit distance.” This suffices to find “the depth” of any bead on any string as the length ratio of the stretch for the bead to the zero depth point to that of the unit to the zero depth point (Koenderink & van Doorn, 2012). The pair of glass panes is a mental synchronization device—Hildebrand used it literally in his sculpturing practice—that “unifies” pictorial space for an observer.

The device is fully idiosyncratic. When different observers view the same picture, they are likely to apply different “gauges” (pane pairs). As a consequence, their depth values may only be compared after a suitable “gauge transformation.” Such gauge transformations appear as *changes of mental viewpoint*. Notice that “mental viewpoint” is not to be understood as a particular point in pictorial or physical space. It refers to the gauge applied by the genesis of visual awareness. The effect is formally somewhat similar to a change of vantage point in physical space, the main difference being that a “change of mental viewpoint” cannot change occlusion relations. The term should be understood appropriately. It makes no sense to directly compare observers, or the same observer at different times, or different methods to quantify depth properties. Such comparisons are void because results are likely to be in different gauges (Koenderink & van Doorn, 2012). The difference may be substantial: A gauge transformation may reveal an insignificant correlation of depths to be highly significant *modulo* such a transformation, thus showing that straight depth comparisons yield a distorted view of the relation between two visual awarenesses. Hildebrand was well aware of this when he remarked that observers are unable to distinguish between sculpture “in the round” and in “low relief” when you put both pieces against a wall at some distance from the observer.

In previous work, we showed that gauge transformations are idiosyncratic, possibly applied in a piecewise fashion and influenced by particular empirical methods (Koenderink et al., 2001, 2012). But although we suggested the term “mental movement” for gauge transformations, we were not certain whether observers could deploy them voluntarily, nor could we be certain whether observers have access to global representations of pictorial relief or only to mutually weakly connected coherent patches. We have addressed the latter question in a related paper (Koenderink et al., 2015); the former, we address here.

## Methods

### Observers and Viewing Modes

In empirical studies on pictorial space, one requires observers that can actually see in pictorial mode. Although this may sound trivial, it is of key importance. Strict enforcement of the scientific method often forces one to use groups of naive observers. One finds that perhaps a quarter of these requires up to two- or fourfold the time required by the others and that their results are very noisy, often hardly showing signs of coherent pictorial relief. Reporting group averages—the obvious solution to avoid having to report the differences—then would seriously misrepresent the actual facts.

Here, we study three observers (the authors) for which we are quite certain that they can see in pictorial mode. However, since we are interested in detecting whether observers can voluntarily adjust their mental viewpoint in the same task on the same picture, we also need participants who are experienced enough to attempt doing this. The authors have years of experience in diverse visual tasks. In the case it turned out that they cannot change their mental viewpoint, we would not have learned much and should perhaps have studied a much larger group. In case it turns out that at least one of them can, we have proof of possibility. In the latter case, one would not logically require a larger group of observers.

Participants were AD, female, aged 67; JK, male, aged 72; and JW, male, aged 51. They used their preferred correction. All participants had normal corrected acuity, binocular stereopsis, and color vision (with JW being perhaps slightly deuteronomalous).

In previous studies, we have frequently found huge differences in mental viewpoints (Koenderink et al., 2001). However, then the instrumental interface or task differed, thus rendering it impossible to conclude that such changes can be brought about voluntarily. In one study, we speculated that observers can look in what might be called “landscape mode” as opposed to what might be called “object mode” (Koenderink et al., 2001). In the former, one conceives of depth as “moving away from the ego-center,” the gauge panes being parallel to the body's frontal plane, whereas in the latter, the gauge panes are adjusted to the “symmetry of the pictorial object.” Of course, these modes are necessarily ill defined. They are mere phenomenological reports. We first spotted such modes in the aforementioned experiment that yielded surprisingly such large inter-observer differences (Koenderink et al, 2001). In that case, a change of task made observers systematically “switch mode”; apparently, they could not help seeing in a particular mode. However, they represent something the participants acknowledge as truthfully representing their experiences, thus they “make sense to them.”

For want of better terms, we refer to these viewing modes as “Mode I” (informally “landscape mode”) and “Mode II” (informally “object mode”). The empirical results will allow us to decide whether viewing in different modes yields different results for single observers and whether results for different observers viewing in the same mode are similar. We are especially interested to see whether observers are able to “switch mode” voluntarily, as the switch in viewing mode was induced by a switch in the task in the aforementioned experiment (Koenderink et al., 2001).

### Presentation and Viewing

Observers viewed monocularly from a distance of 78 cm. They used a chin rest to fix the vantage point.

The stimuli were presented on a DELL U2410f monitor, a 1920 × 1200 pixels (517 × 323 mm) liquid crystal display (LCD) screen, in a darkened room. We used the standard Apple settings for white point and gamma. The stimulus filled the width of the screen. Below was an empty black area, except for a progress counter. A black area on top was used for the user interaction (see later).

### Stimulus and the Geometrical Framework

**Figure 3.**
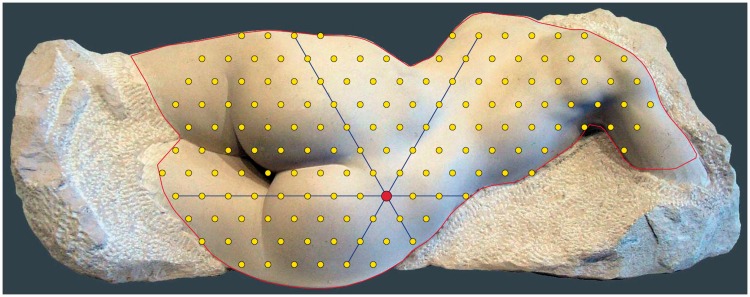
The stimulus with geometrical framework. The stimulus is a photograph of a sculpture described in earlier reports (Koenderink, van Doorn, & Wagemans, 2014; Koenderink et al., 2015). The red contour defines the area of interest. The yellow dots are the vertices of a triangulation of this area. For any vertex (illustrated by the red dot) we define up to three “cross sections.” Each cross section appears in pictorial space as a curve of beads; the task is to reproduce this curve on an abacus-like interface (see text). Notice that most of this geometrical infrastructure remains hidden during the sessions.

In Figure 3, we show the stimulus with the geometrical framework we imposed upon it.

On the picture plane, we defined an area of interest as a connected region that might be expected to be experienced as a smooth pictorial relief. We then defined a discrete triangulation of this area, omitting vertices that would lead to weakly connected subregions. Finally, we defined cross sections as maximum-length collinear subsets of vertices, omitting cross sections of length less than three. Notice that three cross sections in mutually distinct directions (0°, 60°, and 120°) pass through each generic vertex. One boundary vertex supports just two of these cross sections.

There are 157 vertices and 53 sections. The median length of a section is 8 (interquartile range 6–10). Shortest length is 3 (four instances), longest 19 (one instance).

### Sampling and Construction of the Pictorial Relief

**Figure 4.**
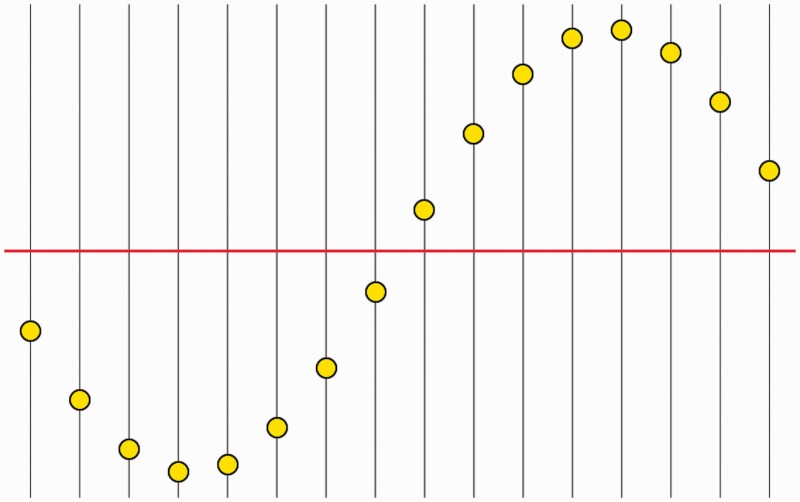
The interface is “a (1 + 1)D abacus,” drawn right above the stimulus picture on the display. The participant reports the cross section in pictorial space, that is current visual awareness, by adjusting the beads (yellow dots). The beads can be grabbed by the cursor and moved along its thread with a mouse drag. Participants could use any order and arbitrarily iterate their settings. The red line is the average of all bead positions; it was kept at the same height on the screen algorithmically, thus forcing the participant to ignore the absolute positions.

The interface is shown in Figure 4. It enables participants to report the cross sections in their pictorial relief (Koenderink et al., 2001). They could use as much time as they thought fit. A keystroke indicating satisfaction then triggered the presentation of the next section. The cross section with vertices upon it was superimposed on the picture, all other vertices, the contour, and so on, being omitted. Participants experience the task as a “natural” one. Cross sections were visited in random order.

A complete session yields a large number of cross sections and for each cross section a number (its length) of relative depth samples. Thus, there are many more depth samples than there are vertices. This allows one to check on the mutual consistency of the samples. We simply construct the surface that best—in the least squares sense—accounts for all samples. The surface is only defined up to a constant, thus we (arbitrarily) constrain its average depth to zero. Then we determine the distribution of deviations from the optimal surface. Combined with the total depth range of the surface, this yields a useful measure of the depth resolution.

In optimizing the “mesh,” we move sections mutually in depth until the depths of the (usually three) samples at any given vertex coincide as closely as possible in the least squares sense. Notice that this procedure might be changed in various ways. For instance, instead of moving the section in depth, one might also apply shears to them. This would undoubtedly improve the fit and might also be defended as reasonable because observers indeed may well change their mental viewpoint when faced with the next section. We decided to use the simplest method though because this proves to be “good enough” and is conceptually simpler in the subsequent analysis.

We consider the surface—referred to as “pictorial relief”—and the remaining deviations, summarized through the depth resolution, as the result of a session. Performing several sessions allows for additional checks of significance, although any such repeat will most likely involve a (perhaps slightly) different gauge.

### Gauge Transformations

Let (*x, y*) denote Cartesian coordinates of points in the picture plane. A “pictorial relief” is a surface that may be described by a depth variation *z*(*x,y*), such that *z*(*x,y*) = 0 describes the nearest pane of the gauge and *z*(*x,y*) = 1, the far pane. What does it mean to apply a “gauge transformation”? Apparently, a transformation like *x*′ = *x, y*′ = *y, z*′ = *A* + *B*·*x* + *C*·*y* + *D*·*z* describes it formally. Notice that the (*x, y*) coordinates remain fixed, thus one has mere shifts of depth beads along their wires. This transformation is formally the group of “proper movements” of “singly isotropic space.” It is a non-Euclidean space that occurs in classical physics (Yaglom, 1979) and geometry (Sachs, 1990; Yaglom, 1979). Because simpler and more symmetric than Euclidean space, it has been proposed as especially suitable to familiarize school children with geometry (Strubecker, 1962^7^).

The shift *A* may be ignored because absolute depth is a nonentity. The parameter *D* describes a depth dilation or contraction. In cases of depth inversion, as spontaneously occurs with the Necker cube or Figure 1(c), it may become negative. If you cannot discriminate sculpture in the round from a low relief, as is common, this *similarity* (formally an “isotropic similarity of the second kind”) comes into play. Observers lacking stereopsis are characterized by *D* = 0 throughout. The parameters (*B, C*) define what would be called a “shear” in Euclidean space. It is formally known as an isotropic *rotation*. It comes into play when you view an en face portrait from an oblique vantage point (Koenderink et al., 2004). It is often convenient to express the magnitude in degrees by taking the arctangent. These angles are in the range ±90°, for isotropic rotations are not periodic as in Euclidean space (Koenderink & van Doorn, 2012; Yaglom, 1979). The reason is simply that no mental movement will let one see the back of the head of a person painted en face.

Notice that the gauge transformations compose an Abelian group (Hilbert & Cohn-Vossen, 1932). One easily checks that there is an identity element (*A* = *B* = *C* = 0, *D* = 1), that the concatenation of two gauge transformations is another gauge transformation (first *A*_1_, *B*_1_, *C*_1_, *D*_1_, followed by *A*_2_, *B*_2_, *C*_2_, *D*_2_ yields *A*_12_ = *A*_2_ + *A*_1_·*D*_2_, *B*_12_ = *B*_2_ + *B*_1_*D*_2_, *C*_12_ = *C*_2_ + *C*_1_*D*_2_, *D*_12_ = *D*_1_*D*_2_), that each transformation has an inverse (*A*′ = −*A*/*D, B*′ = −*B*/*D, C*′ = −*C*/*D, D*′ = 1/*D*) and that the order in which gauge transformations are applied is irrelevant. Thus, the gauge group has the same structure as the group of eye rotations conforming to Listing's Law (Helmholtz, 1867).

This formalism is of crucial importance in virtually any experiment on pictorial space (Koenderink et al., 2011). Suppose we sample depths *z*_*i*_ at locations (*x*_*i*_, *y*_*i*_) in an experiment, say at *i* = 1, 2, …, *N*, for *N* in the order of a hundred or more. Let another observer yield depth samples *z*_*i*_′ at corresponding points. Because the observers are likely to apply different gauges, even when aware of a very similar pictorial object, their depth values *z*_*i*_ and *z*_*i*_′ cannot be directly compared. One may well find a small or even insignificant correlation. A valid comparison requires a gauge transformation in order to perform the comparison in the same gauge. The required transformation can easily be found by way of a multiple regression involving not just the depths but also the (*x*_*i*_, *y*_*i*_) coordinates in the picture plane (Koenderink & van Doorn, 2012). Doing this, it is not uncommon to see the coefficient of determination (*R*^2^) jump from nonsignificant to highly significant (Koenderink et al., 2001, 2012). We show an example later.

We use this method of comparison modulo gauge transformations extensively in this study. The parameters *B, C*, and *D* are of additional interest as they quantify the “difference of mental viewpoint” of the two observers.

## Experiment

Three participants set all cross sections on six different occasions. For each session, all settings were fully randomized. On three of these occasions, they attempted to use “Mode I” viewing, on the others “Mode II” viewing (in randomized order). Participants necessarily attach their own meaning to these modes, although they are able to arrive at mutual agreement in discursive thought.

Participants were free to take their times. Setting a cross section takes about half a minute on the average. The distribution is well described by a gamma distribution with typical parameters 4 (the shape parameter) and 8 s (the scale parameter). Of course, the setting depends upon the length of the cross section. All participants considered the task a “natural” one, although the “drawing” of the cross section requires some self-confidence.

For each session, we constructed the best-fitting surface, essentially a list of depth samples with zero mean. We then transformed the results of each participant from Mode I to a single gauge and determined the median depth per location. This procedure was repeated for Mode II. We determined the depth range of these surfaces as well as the median of the absolute deviations. This yields the depth resolutions. These data were then used as the input to the formal analysis.

### Analysis

Depth ranges are different for the modes. For all observers they are at least twice as large in Mode I as compared to Mode II. The resolutions show a similar pattern. Table 1 shows the median data.

**Table 1.** The Depth Ranges and the Resolutions for All Participants and the Two Modes.

	Mode I range – resolution	Mode II range – resolution
AD	249–70	97–42
JK	312–103	110–41
JW	228–96	114–45

The depth ranges are technically expressed in pixels (the boundary box of the triangulation measures 1205 × 565 pixels) but should be understood as being in arbitrary units. The resolutions are expressed in terms of the number of discriminable depth layers, a dimensionless number. Mode I refers to “landscape mode,” Mode II to “object mode” (see text).

The ranges are much less informative than the resolutions, since their differences can be transformed away by a gauge transformation. The resolutions are well defined, the spread amounting to about 20%. Notice that observers resolve many depth layers in either mode, showing that they indeed possess stereopsis to roughly similar degrees, and that the method is a valid one.

**Figure 5.**
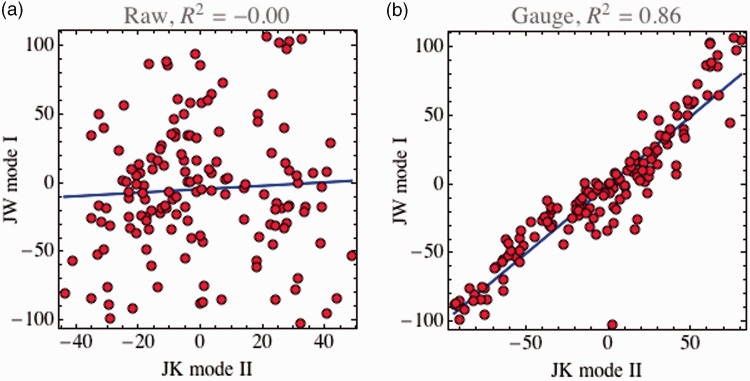
(a) A straight scatterplot of depths values at corresponding points for the case of observer JK in Mode II with observer JW in Mode I. (b) The same data after a suitable gauge transformation. The straight comparison would indicate that the depths for the two cases are essentially uncorrelated. This conclusion is far off the mark though, for in a more suitable gauge the coefficient of determination (adjusted *R*^2^) is as high as .86.

At this point, it might be of interest to illustrate what was said earlier, namely that a sensible comparison of depth values is only possible modulo gauge transformations. Figure 5 shows an instance, the comparison of observer JK in Mode II with observer JW in Mode I. Here, a straight comparison suggests that these cases lead to essentially unrelated data (coefficient of determination slightly negative, close to zero), whereas viewing the data in the right gauge reveals them to be essentially equivalent (coefficient of determination [adjusted *R*^2^] is .86).

For all observers, the straight correlations for a comparison of modes are low, whereas they are much higher when viewed in a suitable gauge (Table 2). This indicates that all participants use a different gauge when viewing in Mode I as compared with viewing in Mode II.

**Table 2.** Coefficients of Determination (adjusted *R*^2^) for the Comparison of Viewing Modes I and II for All Participants.

	I–II raw	I–II gauge
AD	0.28	0.83
JK	0.06	0.87
JW	0.05	0.85

In Table 3, we specify the adjusted coefficients of determination for all cases. In a raw comparison, the correlations are much lower for the comparison of different modes than for same modes. Considered in a suitable gauge, these differences disappear.

**Table 3.** Coefficients of Determination (adjusted *R*^2^) for the Comparison of Raw Depths (Left) and in the Suitable Gauge (Right) for all Pairs of Participants.

	I–I	II–II	I–II	II–I		I–I	II–II	I–II	II–I
AD–JK	.71	.51	.11	.14	AD–JK	.82	.62	.86	.82
AD–JW	.71	.67	.25	.04	AD–JW	.87	.69	.78	.84
JK–JW	.76	.34	.12	.00	JK–JW	.86	.51	.80	.86

*Note*. At left, for the raw depths, the values are high for the same modes and low for the different modes. At right, for the depths in the suitable gauge, this distinction is not apparent. Seen from the right gauge, the viewing modes are not really different.

**Figure 6.**
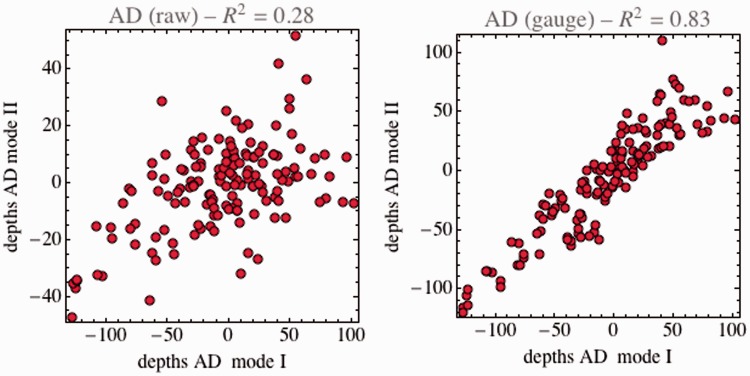
Scatterplots for the depths at corresponding locations obtained in the two viewing modes. This is for participant AD; the plots for the other participants look very similar.

**Figure 7.**
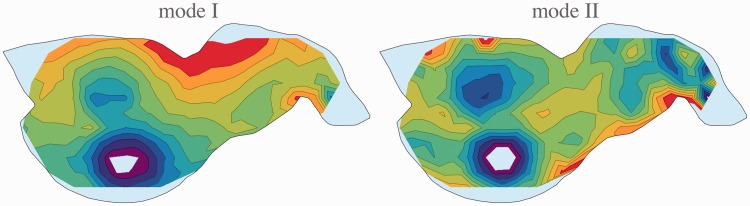
Contour plots of depths obtained in viewing Mode I (left) and Mode II (right). This is for participant AD; the plots for the other participants look very similar. The hue scale runs from blue (near) to red (far).

In Figures 6 and 7, we illustrate the case for participant AD. Similar plots for the other participants look essentially similar. In the raw representation, the correlation for comparisons is low (coefficient of determination .28), whereas it turns out to be quite high (coefficient of determination .83) in a suitable gauge. Notice that the corresponding contour plots for the depth look very different.

The parameters of the corresponding gauge transformation for AD involve a similarity with scale factor 1.46 and a shear of 12.6° in the direction of 2.6° from the vertical in the clockwise direction. We find similar shears for the other participants (20.5° in a direction 0.8° from the vertical in the counterclockwise direction for JK and 15.4° in the direction of 2.1° from the vertical in the counterclockwise direction for JW), but the scale factors vary substantially (1.46 for AD, 1.16 for JK, and 0.46 for JW, a range of 3.2). The shears are just as expected from the—necessarily informal—viewing instructions. We find that the scale factors generally vary idiosyncratically by factors of four or more, so we cannot draw any conclusions from that.

**Figure 8.**
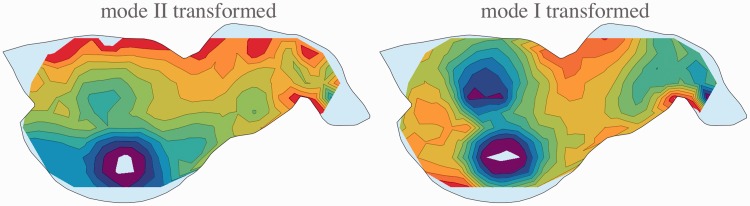
The reliefs shown in Figure 7 transformed to the gauge of the other mode. Note that this neatly reproduces the direct measurements. The reliefs from the two modes are fully equivalent and differ only through some mental movement.

In Figure 8, we show the results for Modes I and II, observer AD, back transformed. One should compare these plots with the ones in Figure 7. It is evident that the reliefs for the two modes are indeed equivalent and can simply be transformed into each other with a suitable gauge transformation.

The changes in mental viewpoint of 13°–21° for the different viewing modes are indeed substantial, but not remarkable. In previous experiments, we have encountered changes up to as much as 70° (Koenderink et al., 2001). These differences may be due to the stimulus, the present picture presenting a sculpture in close to fronto-parallel attitude, whereas in the previous experiment a flattish object was shown as leaning backward (lying on the floor).

## Discussion

There are some interesting facts to glean from this data:

- All observers have good stereopsis in both Mode I and Mode II; observers have similar depth resolution, which renders the common interpretation of their data reasonable.
- Suitable gauge transformations reveal different observers to be essentially similar in both modes.
- Individual observers yield raw depth data that are quite different when they view in Mode I or Mode II.
- Suitable gauge transformations reveal the data of any observer to be essentially independent of mode.
- The necessary gauge transformations indicate shears that neatly reflect the viewing instructions and are remarkably similar for all observers.

Some phenomenological facts that are not revealed by the quantitative data may nevertheless be relevant in attempts to draw conclusions. All participants had a clear preference for one of the two modes and complained that the other required some effort. This may indicate that they had to apply some additional mental transformation. (Not to be confused with Shepard's “mental rotations,” which occur between different physical projections of a given 3D configuration. In our case, all there is, is a single picture.) However, we cannot detect traces of that in the response times, which—given the nature of the task—is perhaps not too surprising. Not all participants had a preference for the same mode. Thus, AD preferred Mode I, whereas participants JK and JW preferred Mode II. We have encountered such different attitudes before in a slightly different setting (Koenderink et al., 2001). In that case, AD and JK were also participants and had clearly distinct preferences. It would appear that there is room for additional research here.

As always in this type of experiment, there remain quantitative differences that appear to be essentially idiosyncratic (Koenderink et al., 2011). Here, we notice appreciable differences in depth range and scale factor in the gauge transformation from Mode I to Mode II. There is no explanation for this, except that the observers are “simply different” in some respects. On the other hand, it can hardly be a coincidence that the observer data correlate highly when considered modulo gauge transformations. Apparently, they are aware of very similar pictorial spaces, which again is best explained by the fact that they happen to look at the same picture.

An issue that often comes up in this kind of work concerns the use of a picture of a human nude (a photograph of a sculpture of a reclining nude female here, a photograph of a female shopwindow mannequin in many of our previous studies). The implicit assumption behind such remarks is that knowledge of the human form places enormous constraints on the perceived depth relationships. This is a common misconception. Knowledge of the human form hardly places any constraint on depth relations as operationally defined by our methods. Few people have any notion of the human figure that goes beyond an ability to name the most obvious members. Training someone to understand the human figure takes years of art school. Many artists keep working on the figure throughout their life, despite the fact that they have picked up the ability to name legs, arms, head, and so forth. Artistic anatomy is not just anatomy. For instance, does the knee belong to the upper or lower leg? The answer does not derive from anatomy but artistic vision: It depends upon the pose and is crucial in drawing. Naive observers are aware that faulty drawings are somehow “wrong,” but at a loss to say why, despite their ability to point out body parts. Muscular bulges more prominent than the nose are often missed, even by art students who have been trained to use their eyes. For most art students, the ability to name a nose is a major obstacle in drawing a face. One object of art education (“learning to see”) is to forget about seeing named parts as “objects.” That nudes are “familiar” objects is somewhat convenient in that we can use names of familiar parts instead of image coordinates, but it in no way implies that the observers would be able to call up depth with their eyes closed. We selected this shape for a variety of technical reasons, but issues of “familiarity” did not play a role in the choice. We are confident that they are irrelevant but if one wanted to study this issue by systematically manipulating the familiarity with the depicted object or even the particular picture, the present methods would allow one to do so.

A similar issue pertains to the level of experience of the participants in this study (the authors), who are not only familiar with the human form, but have also participated in other, time-intensive, perceptual studies employing this picture (Koenderink et al., 2014, 2015). Does this imply that they can clearly visualize the relief with their eyes closed or that the experimental procedure could also be applied effectively to the unseen portions of the nude's body? No. Pictorial relief is strongly dependent on the available stimulus information. In the past, we have also worked on silhouettes and on cartoon drawings without any shading (Koenderink et al., 1996a). Even with highly experienced observers, one notices changes of relief as one goes from silhouette to cartoon, to photograph, and one finds different reliefs for photographs of objects in identical pose but different illumination (Koenderink et al., 1996b). The same holds for occluded parts not represented in the image. In such cases, results are (as expected) idiosyncratic and there is little inter-observer concordance even for experienced observers (Koenderink & van Doorn, 2003). The fact that pictorial relief is subjective and that observers can change their mental viewpoint when performing tasks to measure pictorial relief does not turn it into a “mental imagery” experiment.

## Conclusions

The empirical results allow us to answer our major question in the affirmative: Human observers are to some extent able to assume different mental viewpoints voluntarily. At least the three experienced observers experiencing evident (“paradoxical monocular”) stereopsis could redirect their “mental view direction”(as quantified by the gauge transformations) by up to20°.

Of course, this must necessarily remain a “proof of principle” since we did not study some sizable group of observers. That would involve a major undertaking, since many members selected at random from the population will have none or only weak stereopsis. One would certainly have to select or grade observers first. Even then, it may well be necessary to teach them to become aware—or even accept—the fact that they experience monocular stereopsis. We have met people who apparently experience (so-called paradoxical!) stereopsis, but when learning about the absence of binocular disparity told us “well, flat after all” (Koenderink, 2013). They preferred to ignore an experiential fact on the basis of their discursive knowledge. This is common enough with people who cannot tolerate “spooky” phenomena. Visual artists might be the most useful community, for at least they have learned “how to see” and to accept their visual awareness even when in apparent conflict with their reflective thought. Most naive observers, including vision scientists, are only prepared to do the latter in the case of the familiar “geometrical illusions.” Illusions do not go away when you know them to be illusions.

Stereopsis may conceivably come in different varieties, perhaps similar to the ability of observers to be aware of holistic versus partial Gestalts. As Elkind and Koegler (1964) showed, children see only the parts or the whole (typically the parts), whereas adolescents are able to switch and mature observers are able to experience multiple levels simultaneously. We certainly know of people who do not experience pictorial space and those who are simultaneously aware of both pictorial space and the space they move in (containing both the eye and the picture surface). Perhaps there are also those who need to switch between these modes. Here lies a huge field of scientific endeavor. To the best of our knowledge, the phenomenology has never been mapped out, so this has to remain speculative.

**Figure 9.**
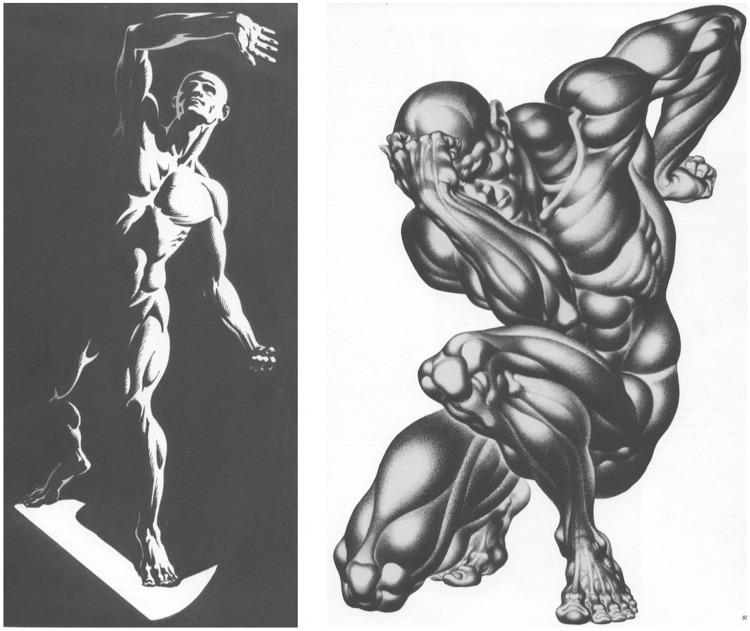
A montage of examples from Hogarth (1981). At left, illumination by a single, directed, distant source ( “Chiaroscuro–One-Third Front,” p. 43). This is perhaps most easily read in something like “object mode.” At right, what Hogarth calls “sculptural light” ( “Complete Forms,” p. 85), which is not illumination at all. This rendering almost forces it to be read in “landscape mode.”

It is also of interest to consider whether people might be able to train the command over their mental viewpoint. One would guess this to be the case and that some variety of yoga training might be indicated. However, the established techniques of the art academies—although aimed at “learning to see”—do not seem to push this point. It seems perhaps related to shading techniques, where shading by light flow is similar to our “object mode,” whereas the sculptural technique of edge shading is like our “landscape mode” (Hogarth, 1981; see Figure 9).

The viewing Modes I and II are partly constrained by the pictorial content. Studying the mental movements for mode changes as a function of such pictorial content; for instance, photographs of objects in different poses might well lead to a refined understanding.

Perhaps fortunately, the bottom line is clear enough: observers are able to deploy their mental viewpoint actively.

## Notes

1. Often called an “ill-posed” problem, which is surely an euphemism.

2. A well-known example of another “mode” is the holistic versus a more analytic mode (Elkind & Koegler, 1964). Confronted with pictures of people constructed from fruits (e.g., a banana for a leg), small children see only the fruits, whereas somewhat older ones see the fruits or the person, but one at a time. Many adults can easily switch or claim to see both simultaneously.

3. The estimate is based on decennia of experience with a few hundred people.

4. The zograscope was just as popular as the stereoscope. Often these instruments were combined into a universal viewing instrument (Koenderink et al., 2013).

5. The affine line has neither an origin nor a unit distance. Ratios of stretches are well defined.

6. Mathematically this construction is known as a “fiber bundle” (Koenderink & van Doorn, 2012).

7. Euclidean geometry is complicated by the fact that the angle measure is periodic, whereas the distance measure is not. That is why there are “parallel” planes, but no “parallel points” in Euclidean space. This is awkward and aesthetically ugly because the underlying projective structure does not differentiate (formally!) between points and planes.
